# Effects of Different Organ Metastases on the Prognosis of Stage IV Urothelial Carcinoma of the Bladder

**DOI:** 10.1155/2022/8594022

**Published:** 2022-11-02

**Authors:** Liang Liu, Haibo Yuan, Qiang Wang, Chuangui Li

**Affiliations:** Department of Urology, Baoding No. 1 Central Hospital, Baoding 071000, China

## Abstract

**Objective:**

To assess the prognosis of stage IV metastatic urinary bladder urothelial carcinoma (UBUC) at initial diagnosis and determine prognostic factors based on distant organ metastasis.

**Methods:**

A retrospective cohort analysis of UBUC was conducted based on the Surveillance, Epidemiology, and End Results (SEER) database. Univariate and multivariate Cox regression analyses were used to determine the variables associated with overall survival (OS). Kaplan–Meier curves were used to compare survival curves among different groups.

**Results:**

A total of 3103 patients with stage IV UBUC were selected for analysis. The number of distant organ metastatic sites independently predicted the OS. The OS was not different in other metastatic sites when bone metastasis was used as a reference (*P* > 0.05). However, the OS was shorter for a single metastatic site (*P* < 0.001) and multiple metastatic sites when metastasis was not used as a reference (*P* < 0.001). Multivariable Cox regression analysis indicated that low survivorship was independently associated with no surgery for the entire cohort and patients with only one metastatic organ. Sex (*P* = 0.019) and grade (*P* = 0.046) were the independent risk factors for patients with only one metastatic organ.

**Conclusions:**

These results show that the prognosis of stage IV metastatic UBUC is not different between any single metastatic organ. The prognosis of stage IV metastatic UBUC depends on the number of distant organ metastasis. This study determined some predictors of survival and thus may help therapists to choose appropriate treatment strategies for metastatic UBUC.

## 1. Introduction

Cancer is the leading cause of death and the major factor limiting life expectancy worldwide. For instance, bladder cancer is the tenth most common cancer globally. About 573,278 new bladder cancer patients were diagnosed in 185 countries in 2020, of which 212,536 cases died [1]. Bladder cancer has several subtypes, including urothelial carcinoma, adenocarcinoma, and squamous cell carcinoma. However, urinary bladder urothelial carcinoma (UBUC) is the most common histological subtype.

Untreated metastatic bladder carcinoma has poor prognoses with a median survival of 3–6 months [2]. Some patterns of bladder carcinoma metastases include lymph node involvement (25.4%) and distant organ metastasis, such as the bone (24.7%), brain (3.1%), liver (18.1%), and lung (19.4%). However, distant organ metastasis is rare [3]. Although organ metastasis is rare in UBUC, it is related to significantly shortened survival [4–6]. Currently, more than 50% of bladder cancer patients are unfit for chemotherapy, according to guidelines. Moreover, the role of surgery as a second-line treatment is unclear [7]. Several studies have revealed that bladder cancer patients with distant organ metastasis at diagnosis can benefit from surgery, especially surgery at the primary site [8, 9].

Moreover, the site and number of organ metastasis can affect the survival of UBUC patients. Therefore, the situation of metastases in new diagnostic cases should be assessed to improve accuracy rates of predicting prognosis and for appropriate management strategy. Some factors, such as distant metastatic sites, histologic types, and neutrophil-to-lymphocyte ratio, are related to the survival of metastatic bladder cancer [10, 11]. Nevertheless, these factors were ignorant of the number of sites for metastasis and lacked prognostic tool for UBUC. Therefore, a new prognostic model is needed to predict survival time in clinics.

A few studies have examined the prognosis significance of the mode of organ metastasis for bladder cancer at the de novo diagnosis. However, most previous studies analyzed a small single-center case series [12–14] or considered bladder cancer as a whole [14, 15]. Therefore, a study of urothelial carcinoma of the urinary bladder with a large population is needed. This study aimed to describe the frequency and pattern of these distant metastases based on the Surveillance, Epidemiology, and End Results (SEER) database. This study also assessed the relationship between different metastasis sites and overall survival (OS) in the de novo diagnosed UBUC patients. Therefore, this study may provide insights into the prognosis of metastatic mode in newly diagnosed UBUC patients, thus helping in choosing a suitable management strategy.

## 2. Materials and Methods

### 2.1. Database and Patient Selection

In this study, data of patients diagnosed with stage IV UBUC between January 1, 2010, and December 31, 2015, were analyzed based on the SEER database. Institutional board review was exempted since the SEER database contains indeterminate patient information. Besides, SEER is a publicly available database and includes 18 population-based cancer registries in the United States.

**Figure 1:**
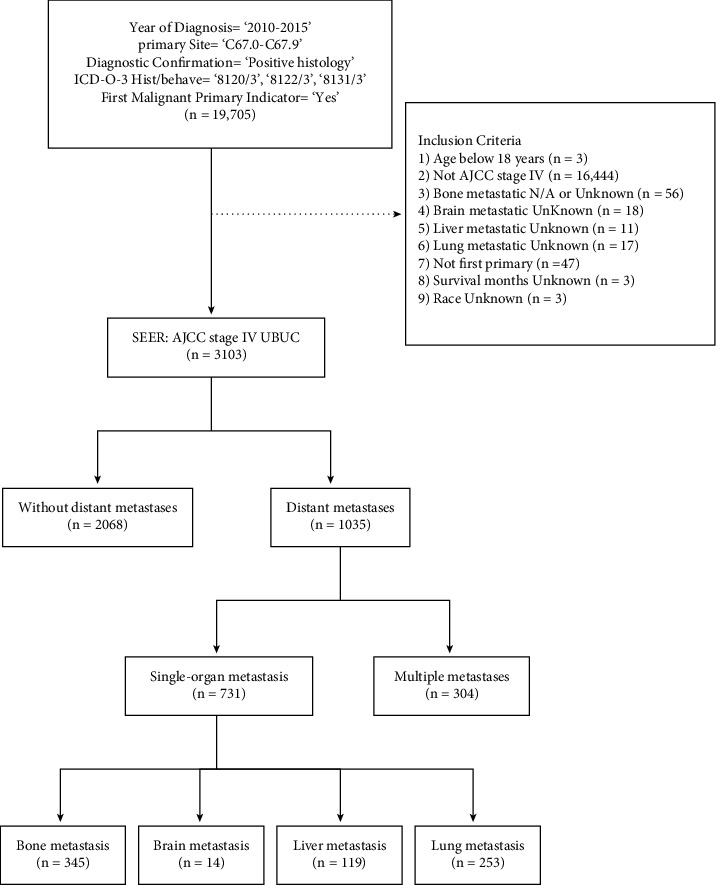
Flow chart of cohort selection of patients.

Inclusion criteria are as follows: (1) Patients diagnosed from 2010 to 2015; (2) Patients aged＞18 years; (3) Patients with positive histology-confirmed UC based on International Classification of Diseases for Oncology, 3rd, edition (ICD-O-3) 8131/3 (Transitional cell carcinoma and micropapillary), ICD-O-3 8122/3 (transitional cell carcinoma and spindle cell), and ICD-O-3 8120/3 (transitional cell carcinoma); (4) Patients with estimated stage IV based on the American Joint Committee on Cancer (AJCC) staging system, 7th, edition; (5) Patients whose primary metastatic site was restricted in the urinary bladder (C67.0–C67.9); (6) Patients with the first or only cancer diagnosis (first positive malignant primary indicator). Patients were excluded if their ethnicity was unknown or there was no information on distant metastatic sites or survival time. SEER*∗*Stat 8.3.9 was used to extract the data (Figure 1).

### 2.2. Study Variables

Basic information (age, sex, and race) of patients were collected. Age was categorized as ＜40 years, ＜60 years, ＜80 years, and ≥80 years. The race was recorded as white, black, and other in the SEER database. Tumor histologic subtypes were recorded as UC (8120/3, 8122/3, and 8131/3) based on ICD-O codes. TNM stage was defined based on the AJCC staging system, 7th edition. Tumor grades were classified as well-differentiated (Grade I), moderately differentiated (Grade II), moderately differentiated (Grade III), undifferentiated/anaplastic (Grade IV), and unknown. The surgical procedure of the primary site was categorized as “none,” “TURB,” “partial cystectomy,” “radical cystectomy,” “pelvic exenteration,” and “other.” Metastasis site at diagnosis was classified as bone (“Mets at DX-bone”), brain (“Mets at DX-brain”), liver (“Mets at DX-liver”), and lung (“Mets at DX-lung”) based on the SEER variables.

### 2.3. Statistical Analysis

SPSS 25.0 was used for all statistical analyses. Categorical data are presented as numbers and percentages. Based on categorical data, chi-square tests were used to determine the statistical difference between conditions. The odds ratio (OR) and 95% confidence interval (95% CI) were determined using logistic regression analysis to ascertain risk factors associated with the likelihood of having distant metastasis at diagnosis. The hazard ratios (HRs) and 95% CI were determined using univariate and multivariable Cox proportional to ascertain risk factors associated with the OS of having distant metastasis at diagnosis. GraphPad Prism 8.0.2 was used to draw a forest map. Kaplan–Meier curve was used to plot the survival curves. The log-rank test was used to compare the survival curves. *P* < 0.05 was considered statistically significant.

## 3. Results and Discussion

### 3.1. Results

#### 3.1.1. The Patient Demographics and Characteristics

A total of 3103 patients were included in the study. The demographic features and clinicopathologic characteristics of UBUC patients in the cohort, including patients with distant metastasis (*n* = 1035, 33.4%), and those without distant metastasis (*n* = 2068, 66.6%) are shown in Table 1. More than half of patients (*n* = 587, 56.7%) with distant metastasis (1035) were 60–80 years, of which most were males (*n* = 757, 73.1%), white (*n* = 878, 84.8%), and had stage T2 (*n* = 456, 44.1%), stage N0 (*n* = 569, 55.0%) and grade III or IV (*n* = 819, 79.1%). For surgery of the primary site, most patients with distant metastasis underwent TURB (*n* = 638, 61.6%), 3.3% underwent pelvic exenteration and 3.0% underwent radical cystectomy. Moreover, more than half of patients (*n* = 1206, 58.3%) without distant metastasis (2068) were 60–80 years, of which most were males (*n* = 1439, 69.6%), white (*n* = 1781, 86.1%), and had stage T3 (*n* = 671, 32.4%), N2 (*n* = 830, 40.1%), and grade III or IV (*n* = 1855, 89.7%). For surgery of the primary site, 638 (30.9%) patients underwent pelvic exenteration, 536 (25.9%) patients underwent radical cystectomy, and 597 (28.9%) patients underwent TURB.

#### 3.1.2. Patient Metastasis Pattern and Incidence of Distant Metastasis

Only brain, lung, bone, and liver metastasis information were obtained from the SEER database. Most patients with distant metastasis had one metastatic site (*n* = 731, 70.6%), 23.4% had two sites (*n* = 242), 5.1% had three sites (*n* = 53), and 0.9% had four sites (*n* = 9). Of the 731 patients with distant metastasis in a single site, 345 (47.2%) patients had bone metastasis, 253 (34.6%) patients had lung metastasis, 119 (16.3%) patients had liver metastasis, and 14 (2.0%) patients had brain metastasis (Table 2).

#### 3.1.3. Risk Factors of Distant Metastasis

**Figure 2:**
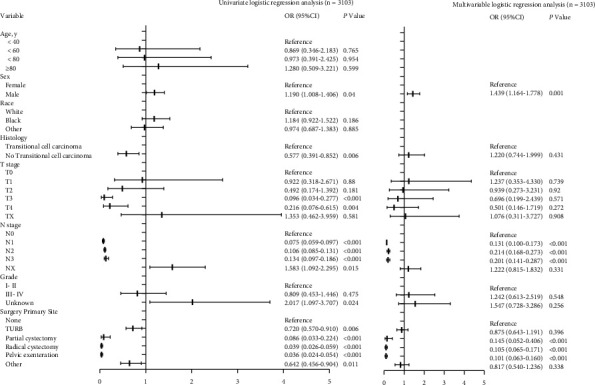
Factors associated with the presence of distant metastases at diagnosis of IV UBUC.

Univariate and multivariable logistic regression analyses (Figure 2) revealed that males had a higher likelihood of distant metastasis at diagnosis than women (OR = 1.439, 95% CI = 1.164–1.778, *P* = 0.001). Surgery of primary site (partial cystectomy vs. none, OR = 0.145, 95% CI = 0.052–0.406, *P* < 0.001; radical cystectomy vs. none, OR = 0.105, 95% CI = 0.065–0.171, *P* < 0.001; pelvic exenteration vs. none, OR = 0.101, 95% CI = 0.063–0.160, *P* < 0.001) was related to a lower likelihood of having distant metastasis. Interestingly, patients with lymph node metastasis were not more likely to have distant organ metastases than lymph node-negative patients (N1 vs. N0, OR = 0.131, 95% CI = 0.100–0.173, *P* < 0.001; N2 vs. N0, OR = 0.214, 95% CI = 0.168–0.273, *P* < 0.001; N3 vs. N0, OR = 0.201, 95% CI = 0.141–0.287, *P* < 0.001).

#### 3.1.4. Univariate and Multivariate Survival Analyses

**Figure 3:**
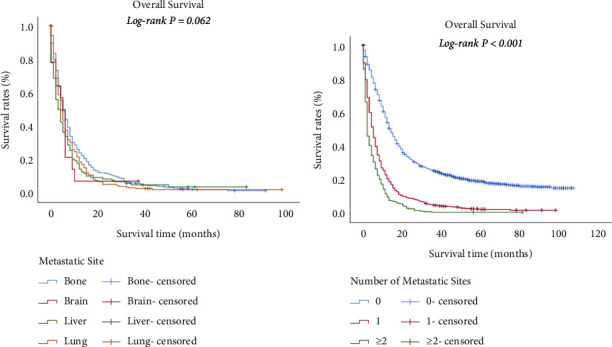
Overall survival of stage IV UBUC based on different distant metastatic sites and number of distant organ metastatic sites. Kaplan–Meier plots indicating overall survival of women with the bone, brain, liver and lung metastases (a) and with 0, 1, 2, and more metastatic sites (b).

**Figure 4:**
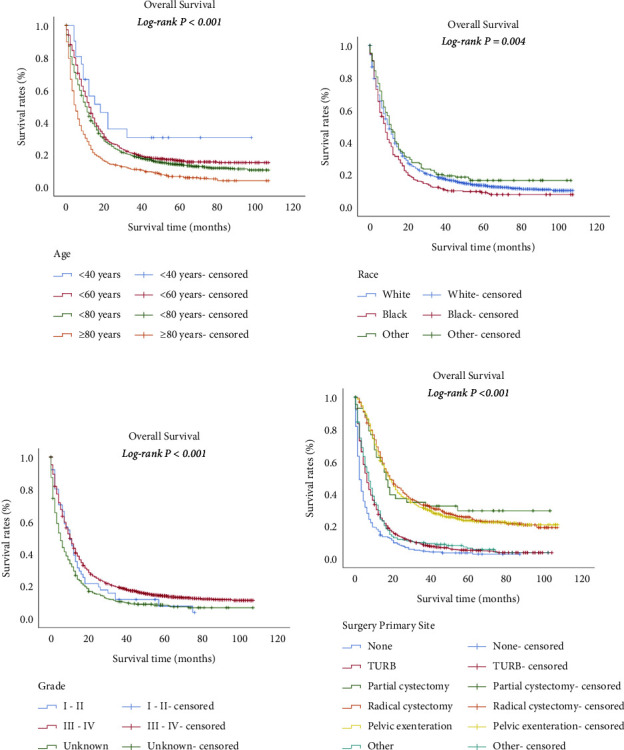
Overall survival of 3103 patients with stage IV UBUC based on demographic and clinical characteristics. Kaplan–Meier plots showing overall survival based on age (a), race (b), grade (c), and surgery of primary site (d) (all *P* < 0.05).

**Figure 5:**
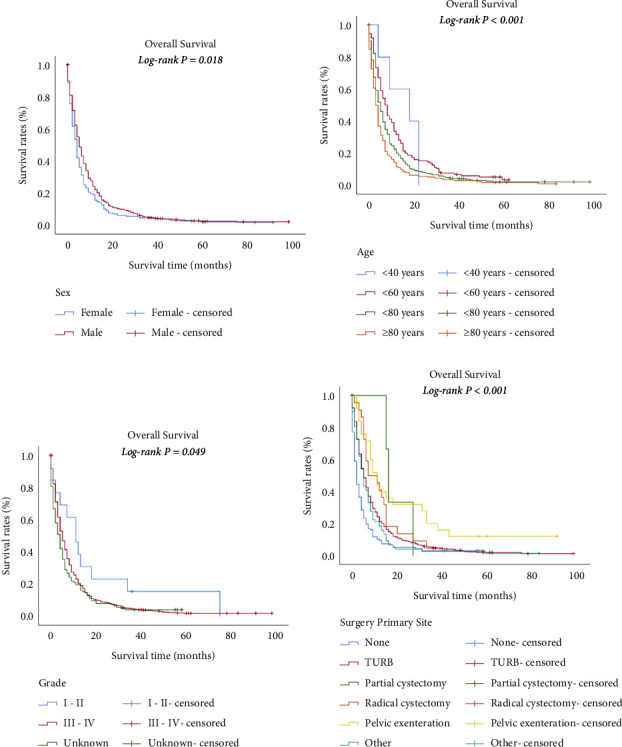
Overall survival of 731 stage IV UBUC patients with only one organ metastatic based on demographic and clinical characteristics. Kaplan–Meier plots showing overall survival based on sex (a), age (b), grade (c), and surgery of primary site (d) (all *P* < 0.05).

The longest survival time of all patients was 107.0 months, which was lessened to 91.0 months for bone metastasis, 37.0 months for brain metastasis, 83.0 months for liver metastasis, and 98.0 months for lung metastasis. The median survival of all patients was 10.0 months, which was lessened to five months for bone or brain metastasis, and four months for liver or lung metastasis. However, statistical comparisons were not significant (*P* = 0.062) (Figure 3(a)). The median survival for the patients without the above organ metastases was 14.0 months, which was lessened to five months for one metastasis site. Patients with two or more metastases sites had the shortest median survival (*P* < 0.001) (Figure 3(b)). The survival estimates stratified by age, race, grade, and surgery of primary site (P < 0.05) for all stage IV UBUC patients are shown in Figure 4. The survival estimates stratified by age, sex, grade, and surgery of primary site (*P* < 0.05) for patients with only one organ metastasis are shown in Figure 5.

**Figure 6:**
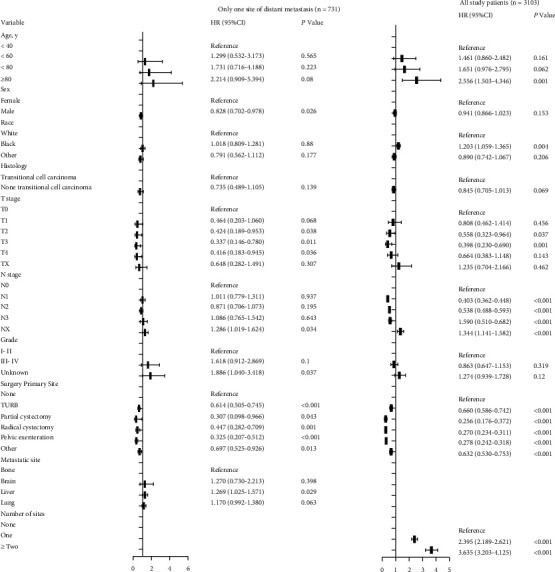
Univariate Cox regression analysis of prognostic factors for overall survival in stage IV UBUC.

Univariate Cox regression analysis of all patients demonstrated that OS was significantly related to age, race, T stage, N stage, surgery of primary site, and number of metastatic sites (Figure 6). However, the OS of 731 patients with one distant organ metastasis was significantly related to sex, T stage, N stage, grade, surgery of primary site, and the number of distant organ metastasis (Figure 6).

**Figure 7:**
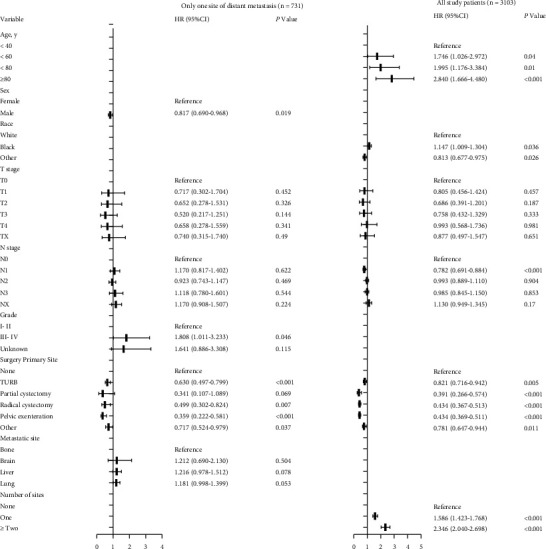
Multivariate Cox regression analysis of prognostic factors for overall survival in stage IV UBUC.

Multivariable Cox regression analysis among all patients indicated that age, race, surgery of primary site, and number of distant metastatic organs could independently predict the OS (Figure 7). The OS decreased with increasing age (＜60 year vs.＜40 year, HR = 1.746, 95% CI = 1.026–2.972, *P* = 0.040; ＜80 year vs.＜40 year, HR = 1.995, 95% CI = 1.176–3.384, *P* = 0.010; ≥80 year vs.＜40 year, HR = 2.840, 95%CI = 1.666–4.480, *P* < 0.001) and the black race (HR = 1.147, 95% CI = 1.009–1.304, *P* = 0.036). Furthermore, low survivorship was independently related to both one metastatic organ (HR = 1.586, 95% CI = 1.423–1.768, *P* < 0.001), and two or more metastatic organs (HR = 2.346, 95% CI = 2.040–2.698, *P* < 0.001) in the entire cohort. Moreover, low survivorship was independently related to no surgery for both the entire cohort and patients with only one metastatic organ. Sex (male vs. female, HR = 0.817, 95% CI = 0.690–0.968, *P* = 0.019) and grade (III- IV vs. I- II, HR = 1.641, 95% CI = 0.886–3.308, *P* = 0.046) were the independent risk factors for patients with only one metastatic organ (Figure 7).

## 4. Discussion

This research analyzed the relationship between the specific metastatic sites and the prognosis of stage IV metastatic UBUC for appropriate treatment strategies.

Of 3103 patients with stage IV UBUC who developed only secondary distant organ metastases, the bone (11.1%) and lung (8.2%) were the common sites at diagnosis, while metastasis in the liver (3.8%) and brain (0.5%) occurred after diagnosis. Besides, 242 (7.7%) patients had two distant organ metastases, (53, 1.7%) had three distant organ metastases, and (9, 0.3%) had four distant organ metastases. Bianchi et al. [3] showed that bone metastasis rate is higher than lung metastasis rate for M1 stage bladder cancer patients. Similarly, this study revealed that bone metastasis was higher than lung metastases for UBUC. Although the information of other metastatic sites was not available due to the limitation of the SEER database, the sites of site-specific distant metastasis were similar to those reported in some previous studies [17–21]. Notably, this study independently assessed UBUC metastasis and had a larger sample size than most previous studies.

In this study, metastasis was an independent prognosis factor for UBUC regardless of the type and number of metastases at UBUC diagnosis. The median survival time among patients without metastasis was 14 months, which was lessened to five months for patients with a single metastatic site, and two months for patients with more than one metastatic site (*P* < 0.001).

Similarly, Chen et al. [11] showed that multiple-site metastasis is independently closely related to a poor prognosis compared with one-site metastasis (HR = 1.428, 95% CI = 1.260–1.618, *P* < 0.001). However, they reviewed cases of bladder cancer (stage I to IV), instead of stage IV metastatic UBUC. Herein, the median survival time for patients with bone or brain metastases and those with liver or lung metastases was only five and four months, respectively (*P* > 0.05).

A recent study showed that bladder cancer patients with liver metastasis have poorer prognoses than those with lung, brain, or bone metastasis. Moreover, the study showed that brain metastasis patients have poor OS than lung metastasis patients [2]. Zhang et al. [22] revealed that patients with bone metastasis have a shorter survival time than those without bone metastasis. This was the first study that compared the prognostic factors of patients with bone metastasis. However, the above study included noninvasive urothelial carcinoma, while our study targeted UBUC patients. Moreover, Zhang et al. [22] only included bladder cancer patients with bone metastasis, while our study targeted all stage IV UBUC patients. Besides, Shou et al. [2] showed that the prognosis of UBUC patients is not significantly different between single site-metastases. However, in our study, although the prognosis of UBUC patients was not significantly different between single specific organ metastases, the number of organ metastases was related to prognosis, suggesting that UBUC metastasis should not be regarded as a single entity. For instance, gender, grade stage, and surgery methods were independent prognosis factors only for single site-metastatic patients, while age, race, lymph node staging, and surgery methods were independent prognostic factors for the whole cohort.

Surgical resection of the primary tumor is a crucial component of the complex remedy of many metastatic tumors [23, 24]. Nevertheless, only a few researches have assessed the impact of surgery on survival outcomes in bladder cancer with organ metastasis [25, 26]. Moreover, only a few researches have evaluated the impact of surgery on survival outcomes of UBUC patients with organ metastasis. Moschini et al. [27] found that surgical resection of the primary tumor can improve the survival time. They also showed that the efficacy of surgical procedures for a primary cancer is associated with the number of metastatic sites.

In the past few years, robot-assisted laparoscopy has gained popularity in urology, gynecology, and general surgery, and is an attractive option for surgeons. Open radical cystectomy with pelvic lymph node dissection is still the gold standard for treating recurrent noninvasive or muscle-invasive bladder cancer, but robot-assisted radical cystectomy is a feasible and relatively safe procedure [28]. Some study by Mastroianni et al. [29, 30] shows that robot-assisted radical cystectomy with intracorporeal urinary diversion perioperative blood transfusion rate was lower than open radical cystectomy (22% vs 41%, *P* = 0.046), and no statistically significant changes were observed in readmission rates and complications of 1 month, 3 months, and 6 months between two groups (All *P* > 0.05). 6 months OS, cancer-specific survival (CSS), and disease-free survival (DFS) have been proved to be statistically comparable for robot-assistedradical cystectomy with intracorporeal urinary diversion and open radical cystectomy. Unfortunately, the median follow-up was only 6 months; therefore, the prognostic factors and long-term survival of robot-assisted radical cystectomy with intracorporeal urinary diversion should be evaluated. Another multi-institutional, multinational study [28] reported the long-term oncologic outcomes of robot-assisted radical cystectomy with totally intracorporeal urinary diversion. A total of 113 cases were enrolled in the study. They found that tumor stage was a significant predictor of survival probabilities (OS: OR = 2.14, 95% CI = 1.46–3.14, *P* < 0.001; CSS: OR = 1.82, 95%CI = 1.3–2.53, *P* < 0.001; RFS: OR = 2.29, 95% CI = 1.58–3.32, *P* < 0.001) for robot-assisted radical cystectomy with totally intracorporeal urinary diversion. They concluded that long-term oncologic outcomes of robot-assisted radical cystectomy with totally intracorporeal urinary diversion appears similar with open surgeries (5-year OS = 54 ± 5%; CSS = 61 ± 5%; and RFS = 58 ± 5%, respectively).

In this research, primary site surgery was associated with a good prognosis, regardless of the presence of metastasis. Patients who did not undergo surgery at the primary site had the shortest median survival time (2 months). However, patients who underwent radical cystectomy and pelvic exenteration had the longest median survival time (18 months), while those who underwent TURB had the shortest (6 months). Furthermore, the median survival time of all metastasis patients with one site was two months. Patients who underwent partial cystectomy had the longest median survival (16 months), while those who underwent other surgeries had the shortest median survival (5 months).

However, this study has some limitations. First, the retrospective research design limits its conclusions, and it is impossible to completely ignore confounding factors, such as smoking history and treatments received. Besides, the SEER database does not have information on chemotherapy and radiation treatment, and thus these variables were not included in this study. Finally, information about cancer recurrence was lacking, and patients who might have distant metastatic recurrence were not considered.

## 5. Conclusions

Detection of distant organ metastasis for UBUC is crucial for appropriate treatment since distant organ metastasis impacts prognosis significantly. Surgery of the primary site is an independent protective factor for UBUC regardless of the presence of metastasis or not. Herein, radical cystectomy and pelvic exenteration significantly prolonged the survival of UBUC patients. The occurrence of distant metastasis and a higher number of metastasis sites for metastatic UBUC were associated with poor OS than patients without metastasis. Bone metastasis was the most common pattern for metastatic UBUC. However, the survival time was not different among single-organ metastases (the bone, brain, liver, and lung). The overall survival time of patients with more than one metastatic site was shorter than that of patients with only one metastatic site and significantly shorter than that of patients without metastasis. Therefore, this study may aid clinicians to better predict the prognosis of stage IV UBUC for the development of individualized treatment.

In summary, the prognosis of stage IV metastatic UBUC was not different among the single-organ metastases. In contrast, the number of distant organ metastasis affected the prognosis of stage IV metastatic UBUC. Therefore, this study provides some predictors of survival, which may help therapists choose appropriate treatment strategies [16].

## Figures and Tables

**Table 1:** Baseline factors associated with the presence of distant metastases at diagnosis of UBUC (*n*, %).

Variable	Distant metastasis (*n* = 1035)	Without distant metastasis (*n* = 2068)
Age, y		
<40	7 (0.7)	14 (0.7)
<60	215 (20.8)	495 (23.9)
<80	587 (56.7)	1206 (58.3)
≥80	226 (21.8)	353 (17.1)
Sex		
Female	278 (26.9)	629 (30.4)
Male	757 (73.1)	1439 (69.6)
Year of diagnosis		
2010	151 (14.6)	370 (17.9)
2011	153 (14.8)	338 (16.3)
2012	188 (18.2)	310 (15.0)
2013	152 (14.7)	342 (16.5)
2014	181 (17.5)	342 (16.5)
2015	210 (20.3)	366 (17.7)
Race		
White	878 (84.8)	1781 (86.1)
Black	108 (10.4)	185 (8.9)
Other	49 (4.7)	102 (4.9)
Histology		
Transitional cell carcinoma	1001 (96.7)	1953 (94.4)
No transitional cell carcinoma	34 (3.3)	115 (5.6)
T stage		
T0	9 (0.9)	6 (0.3)
T1	141 (13.6)	102 (4.9)
T2	456 (44.1)	618 (29.9)
T3	97 (9.4)	671 (32.4)
T4	196 (18.9)	604 (29.2)
TX	136 (13.1)	67 (3.2)
N stage		
N0	569 (55.0)	289 (14.0)
N1	103 (10.0)	694 (33.6)
N2	173 (16.7)	830 (40.1)
N3	56 (5.4)	212 (10.3)
NX	134 (12.9)	43 (2.1)
Grade		
I–II	18 (1.7)	33 (1.6)
III–IV	819 (79.1)	1855 (89.7)
Unknown	198 (19.1)	180 (8.7)
Surgery primary site		
None	227 (21.9)	153 (7.4)
TURB	638 (61.6)	597 (28.9)
Partial cystectomy	5 (0.5)	39 (1.9)
Radical cystectomy	31 (3.0)	536 (25.9)
Pelvic exenteration	34 (3.3)	638 (30.9)
Other	100 (9.7)	105 (5.1)

UBUC, urinary bladder urothelial carcinoma; TURB, transurethral resection of the bladder.

**Table 2:** Frequencies of combination distant metastasis among 3103 patients with stage IV UBUC (*n*, %).

Site of distant metastasis	N	N%
None of distant metastasis	2068	66.6
One site		
Bone	345	11.1
Brain	14	0.5
Liver	119	3.8
Lung	253	8.2
Two sites		
Bone + brain	9	0.3
Bone + liver	57	1.8
Bone + lung	93	3.0
Brain + liver	1	0.0
Brain + lung	10	0.3
Liver + lung	72	2.3
Three sites		
Bone + brain + liver	2	0.1
Bone + brain + lung	3	0.1
Bone + liver + lung	44	1.4
Brain + liver + lung	4	0.1
Four sites		
Bone + brain + liver + lung	9	0.3

UBUC, urinary bladder urothelial carcinoma.

## Data Availability

The data from the present study are available in the Surveillance, Epidemiology, and End Results, https://seer.cancer.gov.

## Abbreviations and Acronyms

UBUC: Urinary bladder urothelial carcinoma
SEER: Surveillance, Epidemiology, and End Results
OS: Overall survival
ICD-O-3: International Classification of Diseases for Oncology, 3rd edition
AJCC: American Joint Committee on Cancer
OR: Odds ratio
95% CI: 95% confidence interval
HR: Hazard ratio
CSS: Cancer-specific survival
DFS: Disease-free survival
RFS: Recurrence-free survival
